# The Eutopic Endometrium Proteome in Endometriosis Reveals Candidate Markers and Molecular Mechanisms of Physiopathology

**DOI:** 10.3390/diagnostics12020419

**Published:** 2022-02-06

**Authors:** Loren Méar, Emmanuelle Com, Khadija Fathallah, Laetitia Guillot, Régis Lavigne, Blandine Guével, Arnaud Fauconnier, François Vialard, Charles Pineau

**Affiliations:** 1 Univ Rennes, Inserm, EHESP, Irset (Institut de Recherche en Santé, Environnement et Travail)—UMR_S 1085, CEDEX, 35042 Rennes, France; loren.mear@gmail.com (L.M.); emmanuelle.com@univ-rennes1.fr (E.C.); laetitia.guillot@univ-rennes1.fr (L.G.); regis.lavigne@inserm.fr (R.L.); blandine.charroy@univ-rennes1.fr (B.G.); 2 Protim, Univ Rennes, Biosit–UMS 3480, US-S 018, CEDEX, 35042 Rennes, France; 3 UVSQ, INRAE, BREED, Université Paris-Saclay, 78350 Jouy-en-Josas, France; 4 Ecole Nationale Vétérinaire d’Alfort, BREED, 94700 Maisons-Alfort, France; 5 Department of Obstetrics and Gynecology, CHI de Poissy, St. Germain en Laye, 78303 Poissy, France; khadija.fathallah@gmail.com (K.F.); Arnaud.Fauconnier@ght-yvelinesnord.fr (A.F.); 6 EA7325-RISQ, UFR des Sciences de la Santé Simone Veil, 78180 Montigny le Bretonneux, France; 7 Genetics Federation, CHI de Poissy, St. Germain en Laye, 78303 Poissy, France

**Keywords:** endometriosis, biomarkers, proteomics

## Abstract

Endometriosis is a common chronic gynaecological disease causing various symptoms, such as infertility and chronic pain. The gold standard for its diagnosis is still laparoscopy and the biopsy of endometriotic lesions. Here, we aimed to compare the eutopic endometrium from women with or without endometriosis to identify proteins that may be considered as potential biomarker candidates. Eutopic endometrium was collected from patients with endometriosis (n = 4) and women without endometriosis (n = 5) during a laparoscopy surgery during the mid-secretory phase of their menstrual cycle. Total proteins from tissues were extracted and digested before LC-MS-MS analysis. Among the 5301 proteins identified, 543 were differentially expressed and enriched in two specific KEGG pathways: focal adhesion and PI3K/AKT signaling. Integration of our data with a large-scale proteomics dataset allowed us to highlight 11 proteins that share the same trend of dysregulation in eutopic endometrium, regardless of the phase of the menstrual cycle. Our results constitute the first step towards the identification of potential promising endometrial diagnostic biomarkers. They provide new insights into the mechanisms underlying endometriosis and its etiology. Our results await further confirmation on a larger sample cohort.

## 1. Introduction

Endometriosis is a common gynaecological oestrogen-dependent inflammatory disorder that affects from 4 to 10% of women of reproductive age (i.e., 176 million women worldwide) [1,2]. The disease is classically defined by the development of endometrium outside the uterine cavity in three main forms: superficial endometriotic implants, endometriomas (ovarian cysts), and deep infiltrating lesions [3]. Although first described more than a century ago [4], endometriosis remains an enigmatic disease in terms of its physiopathology. A number of theories can explain the emergence of endometriosis but none can fully explain both the different forms and the location of the ectopic endometrium [5,6]. This topic is still a subject of debate, and there is no consensus among specialists [7]. The widely accepted theory, proposed by Sampson in the 1920s, is based on the migration of a portion of menstrual debris into the peritoneal cavity via the Fallopian tubes [8,9]. This would be possible through retrograde menstruation, and the theory is supported by several epidemiological and anatomical facts [10,11]. 

Symptoms associated with endometriosis vary widely between women. They mainly include chronic pain and/or infertility and/or fatigue [2,12]. However, such symptoms may arise due to problems other than endometriosis. This lack of symptom specificity is one of the reasons it can take several years before a diagnosis can be established, i.e., 8 years in the U.K [13], 10.5 years in Austria and Germany [14] and 7.4 years in the Netherlands [15]. However, the principal explanation for such a delay is that the gold standard for the diagnosis of endometriosis is still the visualization of lesions by surgery-laparoscopy, followed by histological analysis of the collected biopsies [16,17,18]. Indeed, there is no reliable minimally or non-invasive test for the screening of endometriosis [5,16,19,20,21,22,23,24]. Non-surgical diagnosis is still a challenge and the development of an accurate non-invasive diagnostic test for endometriosis is considered a priority [17].

Proteomics technologies provide promising opportunities to tackle the field of endometriosis, both for understanding the physiopathology of the disease and for the discovery of potential diagnostic markers or therapeutic targets [25,26,27]. Several proteomics studies have been published on endometriosis using a wide variety of techniques, such as 2D-PAGE [28], MALDI-TOF-MS [29,30], SELDI-TOF-MS [31,32], and LC-MS/MS [33,34,35]. Various biological samples were analysed in these studies, including eutopic endometrium [29,31,32,35,36], urine [30,33], serum [35], peritoneal fluid [28], and cervical mucus [34]. 

The results obtained in large-scale proteomics experiments can be affected by the varying severity and different forms of the disease, the intake of hormones, and the phase of the menstrual cycle, as well as the inclusion and exclusion criteria of the control population, thus resulting in high sample heterogeneity [16,37]. A harmonization project for endometriosis studies was therefore conducted by the World Endometriosis Research Foundation, for data collection, processing, and storage [38,39,40,41]. Its main objective was to minimize the heterogeneity and bias between studies. Unsurprisingly, the proteins highlighted by these previous proteomics studies and presented as potential biomarkers were neither further studied nor validated as clinically usable endometriosis markers by orthogonal approaches [23,24,42]. Moreover, the results of these studies could not be reproduced by other groups.

Here, we demonstrate the potential of a differential label-free LC-MS/MS approach for the large-scale comparison of protein expression in the eutopic endometrium at the mid-secretory phase of the menstrual cycle between endometriosis patients and women without endometriosis, confirmed by laparoscopy. We identified several proteins that could be considered as potential biomarker candidates of endometriosis and which merit further in-depth studies.

## 2. Materials and Methods

### 2.1. Sample Collection

Nine women were recruited from the Obstetrics and Gynecology Department of the Centre Hospitalier Intercommunal de Poissy (France) between May 2015 and November 2017. The study was conducted in accordance with the Declaration of Helsinki, and approved by the ethical committee “Comité de Protection des Personnes” (CPP) Ile de France XI (78105 St Germain en Laye, No. 14 083). 

For all patients with endometriosis (aged from 24 to 38), the diagnosis of endometriosis was confirmed clinically, endoscopically, by exploratory laparotomy or transabdominal hysterectomy, and histologically. The control group consisted of five women of reproductive age (aged from 23 to 46) without any malignant disease, hydrosalpinx, cyst, or other visible lesions by ultrasound, and without endometriosis, which was confirmed by surgical exploration.

No women received hormone treatment and all laparoscopies were performed between days 19 and 24 of their menstrual cycles, corresponding to the putative window of implantation during the secretory phase (Table 1). At the end of the surgical procedure, an fendometrium biopsy was performed by the surgeon with an outpatient endometrial sampling device (Cornier Pipelle^®^) for optimal repeatability of sample collection. Endometrial tissues were placed in microtubes and frozen in liquid nitrogen directly in the operating room and stored at −80 °C until protein extraction.

### 2.2. Sample Preparation 

Frozen human endometrium from four women with endometriosis and five women without were ground to a fine powder in liquid nitrogen and resuspended in lysis buffer (20 mM Hepes pH 7.4) supplemented with a protease inhibitor mix (1 mM EDTA, 0.5 mM dithiothreitol (DTT), 1 mM 4-(2-Aminoethyl)benzenesulfonyl fluoride hydrochloride (AEBSF), and 10 μM L-trans-Epoxysuccinyl-leucylamido(4-guanidino)butane (E64)). This suspension was sonicated on ice: 12 pulses, for 10 s, with 30 s in between. After centrifugation (30 min at 15,000× *g* at 4 °C), the supernatant, enriched in soluble proteins, was frozen at −80 °C. A buffer composed of 8 M urea, 30 mM Tris, and 4% CHAPS, supplemented with the same protease inhibitor mix as above, was used to re-suspended the remaining pellet containing the insoluble fraction. After sonication, the lysates were frozen overnight at −20 °C. Finally, after thawing, CHAPS-soluble lysates and the soluble protein-enriched supernatant were centrifuged at 105,000× *g* at 4 °C for 1 h. Supernatants were recovered and pooled (enriched in soluble proteins with enriched CHAPS-soluble proteins) for each sample. The pellets were discarded. The protein concentration was determined using a Bradford colorimetric assay (BioRad, Marnes-la-Coquette, France), according to the manufacturer’s instructions, and the protein extract was stored at −80 °C until further analysis.

### 2.3. Protein Extraction, Digestion, and Liquid Chromatography—Tandem Mass Spectrometry (LC-MS/MS) Analyses 

Protein digestion was performed as previously described (42). Briefly, aliquots of protein extract were diluted in 8 M Urea, 0.1 M ammonium bicarbonate, pH 7.8. Then, proteins were reduced with 700 mM DTT and incubated at 37 °C for 30 min. Protein alkylation was performed by incubating the proteins in 135 mM iodoacetamide for 30 min in the dark at room temperature. Finally, a 0.4-μg/μL trypsin solution (modified, sequencing grade, Promega, Charbonnières, France) and 100 mM ammonium bicarbonate were added to each sample and they were incubated overnight at 37 °C. The tryptic digested samples were then desalted using 100 mg Sep-Pak tC18 reverse phase cartridges according to the manufacturer’s instructions. After elution, tryptic peptides were evaporated in a vacuum centrifuge and stored in 100 μL 0.1% formic acid at −80 °C. 

Mass spectrometry analyses were carried out with a nanoflow high-performance liquid (HPLC) chromatography system (NanoElute; Bruker Daltonik GmbH, Bremen, Germany) connected to a TimsTOF Pro mass spectrometer (Bruker Daltonik GmbH, Bremen, Germany) equipped with a CaptiveSpray ion source (Bruker Daltonik GmbH, Bremen, Germany). The HPLC system combined a compact and dedicated design autosampler, kept at 4 °C, large volume single stroke piston pumps with a solvent degasser, and a novel valve solution that allowed direct infusion for fast and highly reproducible sample analyses. The mobile A (0.1% formic acid, 98% H_2_O MilliQ, 2% acetonitrile (*v*/*v*)) and B (0.1% formic acid, 100% acetonitrile, (*v*/*v*)) phases for HPLC were delivered by the NanoElute system. Approximately 200 ng of each tryptic peptide sample was separated on an analytical column (75 µm × 250 mm Odyssey column packed with C18 beads with a particle size of 1.6 µm and a pore size of 120 Å; Ion Optics Pty Ltd., Parkville, Australia) at a flow rate of 400 nL/min at 50 °C. We ran a 120 min gradient from 2–15% buffer B for the first 60 min, 25% buffer B from minutes 60 to 90, 37% buffer B from minutes 90 to 100, and 95% buffer B from minutes 100 to 110. Finally, the column was washed with 95% buffer B for 10 min before re-equilibration and the loading of the next sample. The temperature of the separation column was controlled using a Sonation nanoLC column oven. Peptides were detected by directly eluting them from the HPLC column into the electrospray ion source of the mass spectrometer. A voltage of 1.4 kV was applied to the vacuum capillary inlet, whereas the sprayer was kept at ground. The temperature of the ion transfer capillary was set at 180 °C. 

Experiments were performed using nitrogen as a bath gas at room temperature and the gas flow velocity was kept constant by regulating the pressure at the inlet and outlet of the TIMS cartridge. Ions were accumulated for 114 ms and mobility separation was achieved by ramping the entrance potential from −160 V to −20 V within 114 ms. MS mass spectra were acquired with an average resolution of 60,000 FWHM (mass range of from 100 to 1700 *m*/*z*) using the parallel accumulation serial fragmentation (PASEF) method. Peptide ions were separated using trapped ion mobility spectrometry, eluted in 100 ms, and detected in the QTOF, generating the TIMS MS heat map. Ions were thus accumulated and trapped according to their mobility. A stepwise decrease in the electrical field permits serial elution of separated ions, which were then transferred to the QTOF. Using the parallel accumulation operation mode, ions were eluted from the second part of the TIMS device while the next series of ions were being introduced into the first part. For the acquisition of PASEF MS/MS spectra, the same TIMS separation was used with the quadrupole isolating a certain ion species only during its mobility elution time and immediately shifting to the next precursor. Parent and fragment spectra were then aligned by mobility values.

Precursor *m*/*z* and mobility information was first derived from full scan TIMS-MS experiments (with a mass range of 100 to 1700 *m*/*z*) to enable the PASEF method. The resulting quadrupole mass, collision energy, and switching times were automatically transferred to the instrument controller as a function of the total cycle time. The quadrupole isolation width was set to 2 and 3 Th, and, for fragmentation, the collision energies were tuned between 31 and 52 eV, depending on the precursor mass and charge. TIMS, MS operation, and PASEF were controlled and synchronized using the control instrument software otofControl 5.1 (Bruker Daltonik GmbH, Bremen, Germany). LC-MS/MS data were acquired using the PASEF method with a total cycle time of 1.31 s, including 1 TIMS MS scan and 10 PASEF MS/MS scans. The 10 PASEF scans (100 ms each) contained an average of 12 MS/MS scans per PASEF scan. In addition, the most abundant precursors which might have been sequenced in previous scan cycles were dynamically excluded from re-sequencing. The acquisition of the MS/MS mass spectra with the TIMS TOF Pro was also performed with an average resolution of 50,000 FWHM (mass range of 100 to 1700 *m*/*z*). 

### 2.4. MS/MS Data Analysis 

Ion-mobility resolved mass spectra, nested ion mobility vs. m/z distributions, and summed fragment ion intensities were extracted from the raw data file using DataAnalysis 5.1 (Bruker Daltonik GmbH, Bremen, Germany). Signal-to-noise ratios were increased by summations of individual TIMS scans. Mobility peak positions and peak half-widths were determined based on extracted ion mobilograms (±0.05 Da) using the peak detection algorithm implemented in the DataAnalysis software. Feature detection was also performed using DataAnalysis 5.1 software and the results were exported in .mgf format. Peptide identification was then performed with Mascot version 2.5.1, applying the previously described search parameters and using its automatic decoy database search to calculate the false discovery rate (FDR) [43,44]. MS/MS spectra were searched against the UniProt Homo sapiens reference proteome database (UP000005640, release 20 June 2018; 21,044 sequences which contain canonicals). Enzyme selectivity was set to full trypsin with one missed cleavage allowed. Selected modifications were carbamidomethylation of cysteines, variable oxidation of methionine, and variable deamidation of asparagine and glutamine.

Proline Studio software (version 1.6.1) was then used for validation and spectral count comparison as previously described [45]. Identified peptides were validated with a peptide rank of 1 and filtered based on Mascot score values to obtain a false discovery rate (FDR) of 1% at the peptide level. For each identified and validated protein, weighted spectral counts were calculated as suggested in Abacus [46], in which shared peptides were combined and weighted according to the associated protein sets. 

### 2.5. Statistical Analysis and Data Mining 

Significant differences between the two groups (Endometriosis vs. Control) were detected by applying a beta-binomial test, implemented in Proline studio, on the weighted spectral counts for each sample. Proteins were considered to be differential when the *p*-value of the beta-binomial test was significant (−log10(fbinomial *p*-value) ≥ 1.3) and the difference at least two-fold.

Principal component analysis (PCA) was performed in R (version 3.5.1 CRAN [47]) using the FactoMineR package [48]. PCA allowed us to quickly summarize and visualize the data set in a lower dimension. Two PCA graphs were obtained, one based on all the identified proteins and the second one only with the differentially expressed proteins. 

Visualization of the proteins of interest into pathways was performed using the KEGG mapper tool available at The Kyoto Encyclopedia of Genes and Genomes (KEGG) (https://www.genome.jp/kegg/tool/map_pathway2.html, accessed on 16 December 2021). Gene ontology term classification and enrichment analysis were performed on Proteomics Research Environment (ProteoRE), a Galaxy toolshed [49]. A comparison with previously published data from a case/control proteomic study of eutopic endometrium and serum during the proliferative phase [35] was performed and Venn diagrams were obtained for shared up- and down-regulated proteins [50].

## 3. Results

A total of nine women of reproductive age (i.e., five controls and four women with endometriosis) were recruited. The endometriosis and control groups did not differ significantly in terms of the age of the women (mean ± SD age of 35.0 ± 8.8 and 29.8 ± 5.9, respectively) in a *t*-test (*p* > 0.05). 

**Figure 1:**
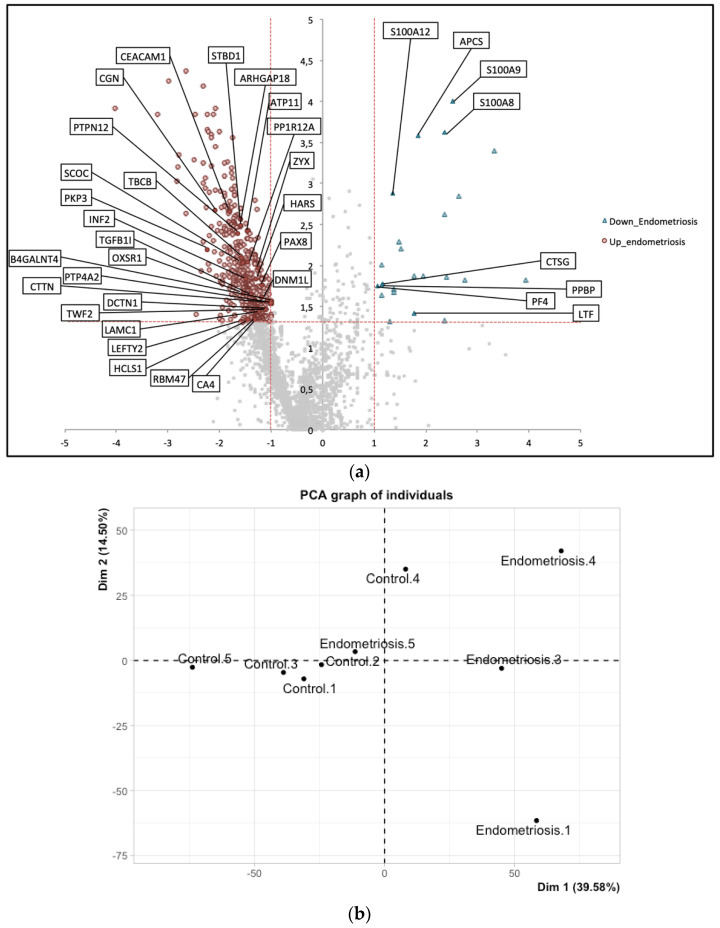

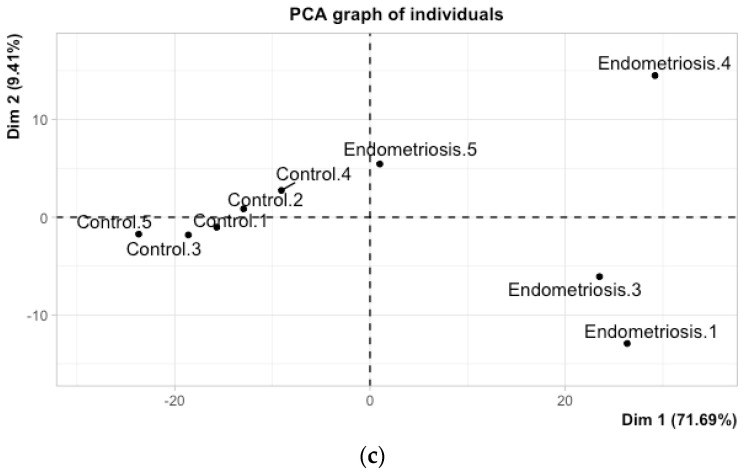
(**a**) Volcano plots illustrate differentially abundant proteins between the eutopic endometrium from control and endometriosis patients. The log10 (binomial *p*-value) is plotted against the log Ratio. The non-axial vertical lines denote a ± two-fold change while the non-axial horizontal line denotes *p* ≥ 1.3, which was our significance threshold (prior to logarithmic transformation). Proteins upregulated with a fold change ≥ 2 and *p* < 0.05 are depicted in red circles and those downregulated with a fold change ≥ 2 and *p* < 0.05 are shown in blue triangles. The proteins shown in grey were not found to differ significantly between the eutopic endometrium of controls and that of endometriosis patients; (**b**) Principal Component Analysis (PCA) applied to all samples based on the expression data of all identified proteins, i.e., fives controls and four endometrioses; (**c**) PCA applied to all samples based on the expression data of the 543 differential proteins.

In total, 5301 proteins were confidently identified in the entire set of eutopic endometrium samples. The control group seemed highly homogenous but this homogeneity could also result from a type I statistical error due to the size of the cohort. Among the 5301 proteins, 543 were identified as differentially expressed at the tissue level with 519 proteins upregulated and 24 downregulated in endometriosis samples relative to normal tissues (Figure 1a). Results of the differential analysis are presented in Supplemental Table S1. As shown in the PCA graph (Figure 1c), this signature of 543 differential proteins allowed us to separate the controls (C1, C2, C3, C4, and C5) from the endometriosis samples (E1, E3, E4, and E5).

**Figure 2:**
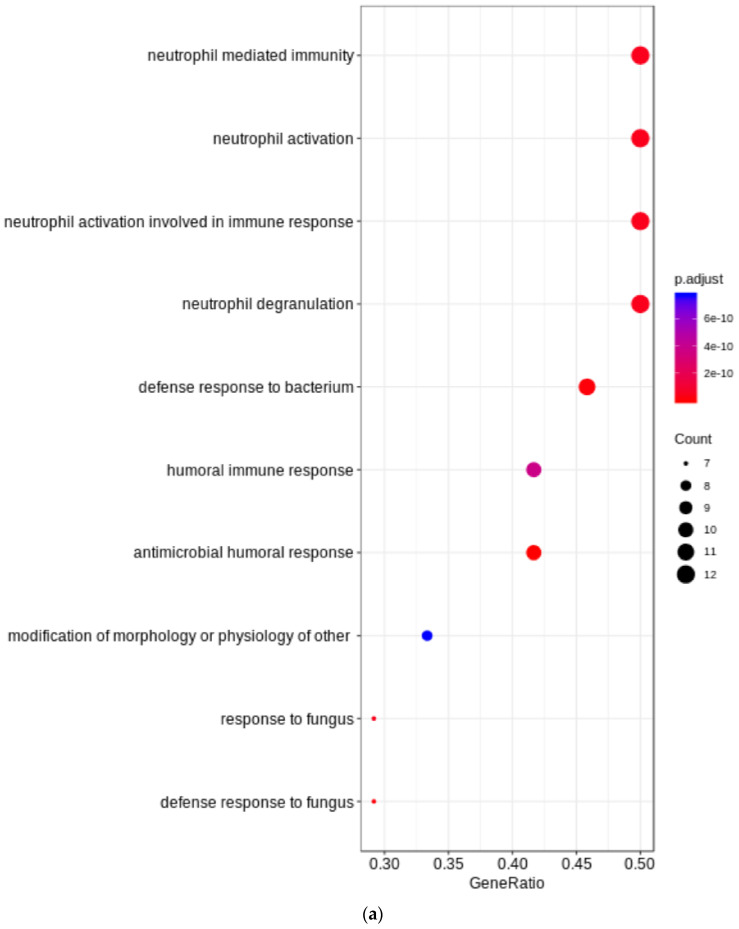

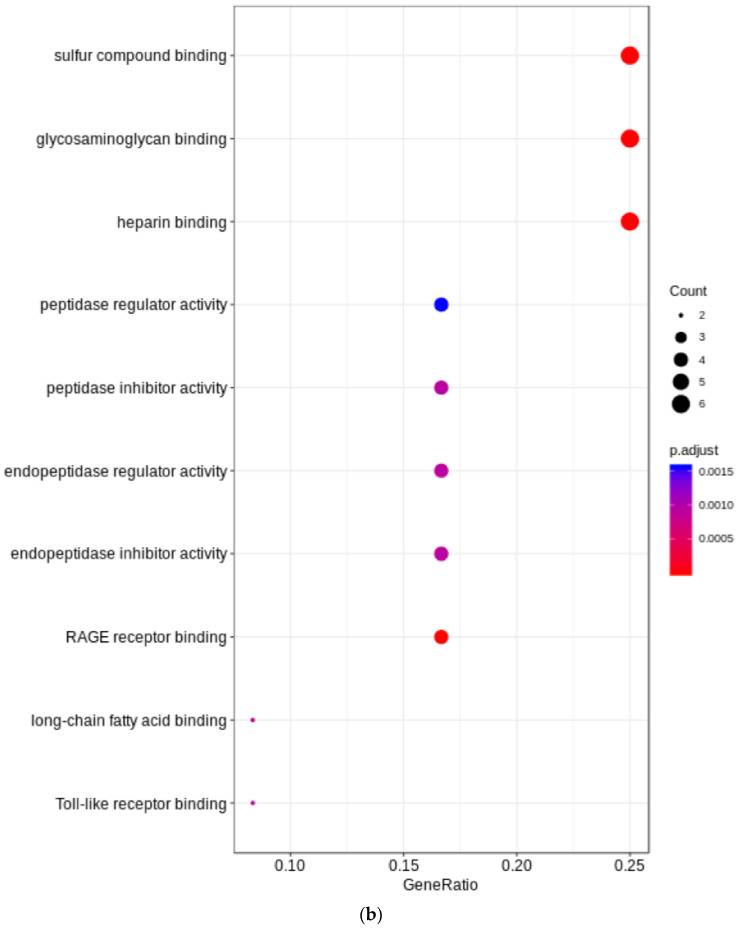
(**a**) Gene ontology biological process and; (**b**) biological function enrichment for downregulated proteins in the eutopic endometrium during the secretory phase (n = 24) of women with endometriosis.

Gene ontology enrichment was not relevant for the upregulated proteins in the endometriosis samples (data not shown), but we observed an enrichment of proteins involved in immune-system processes among the downregulated proteins (Figure 2) (n = 15).

**Figure 3:**
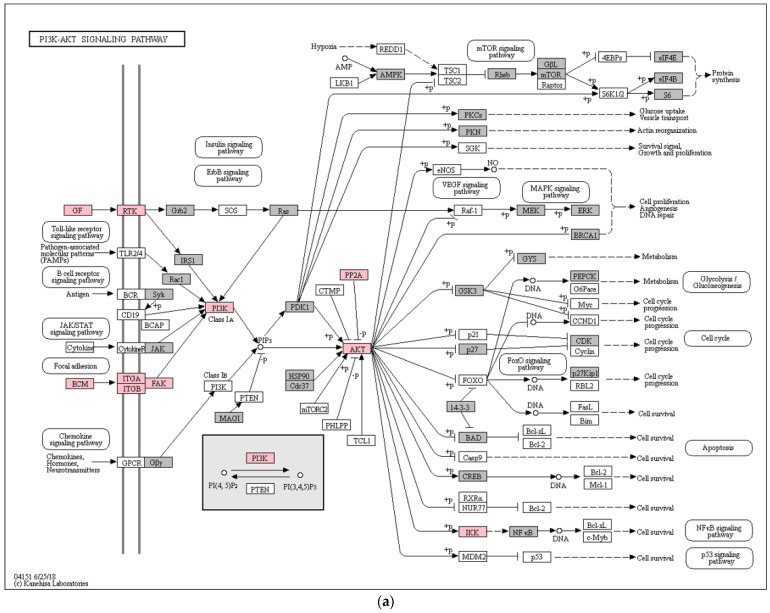

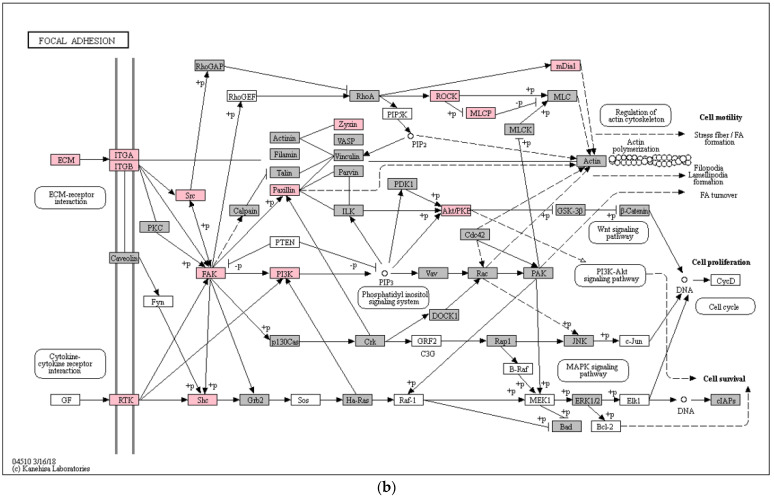
KEGG signaling pathways enriched with identified proteins (grey) and upregulated proteins in endometriosis (pink) (**a**) PI3K-AKT signaling pathways (hsa:04151); (**b**) Focal adhesion pathway (hsa:04510).

KEGG pathway enrichment analysis highlighted two main signaling pathways: PI3K/AKT (21 of 348; Figure 3a) and focal adhesion (23 of 197; Figure 3b), but only for upregulated proteins. 

**Figure 4:**
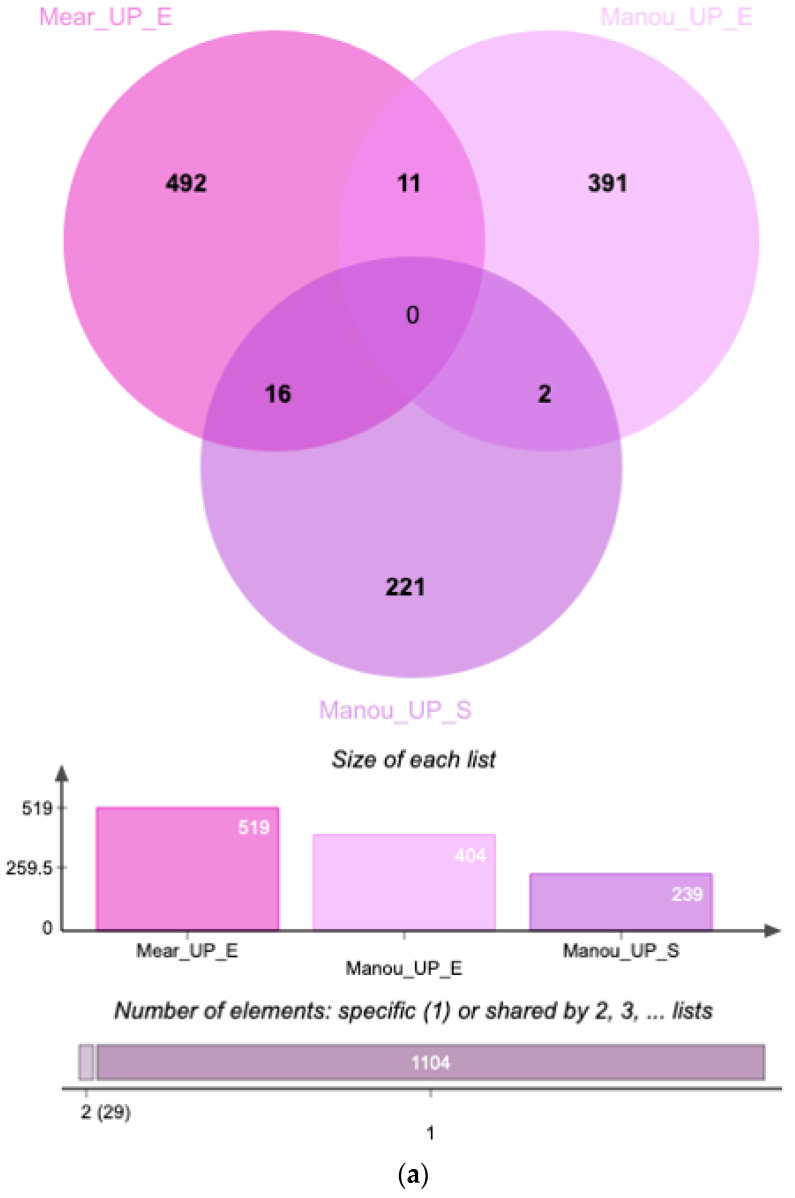

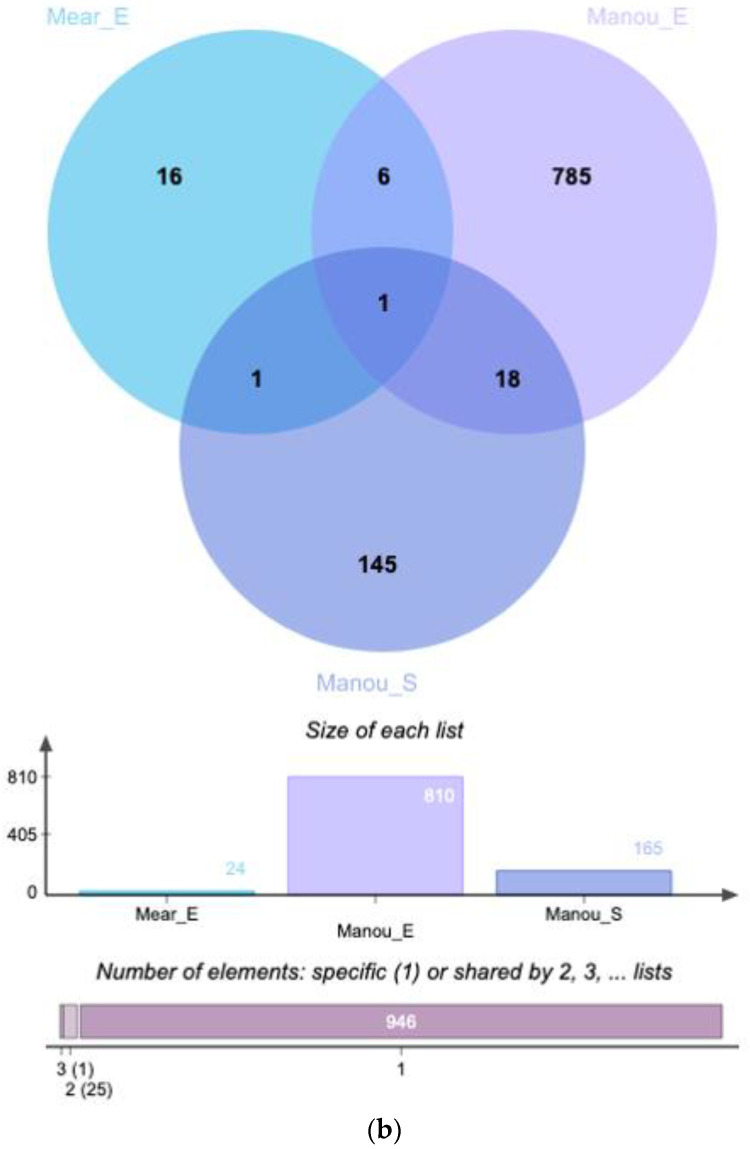
Venn Diagrams (**a**) Upregulated proteins from our dataset (Mear_UP_E) and those of the eutopic endometrium (Manou_UP_E) and serum (Manou_UP_S) of study of Manousopoulou et al.; (**b**) Downregulated proteins from our dataset (Mear_E) and the eutopic endometrium (Manou_E) and serum (Manou_S) of the study of Manousopoulou et al.

Comparison and integration of our data with those from Manousopoulou et al. [35] allowed identification of a putative set of endometrial and serum markers (Figure 4). Indeed, 11 proteins appear to be upregulated in eutopic endometrium during both the proliferative and secretory phases (Table 2, Figure 4a).

Among the 519 upregulated proteins identified in the eutopic endometrium in the secretory phase, 16 were also overexpressed in the serum of women with endometriosis (Figure 4a). There were no upregulated proteins in common between serum and eutopic endometrium in the proliferative and secretory phases. A total of Six of the 24 proteins defined as downregulated by the various analysis were downregulated in eutopic endometrium, regardless of the phase of the menstrual cycle, and one was also under-expressed in the serum of women suffering from endometriosis (Table 2, Figure 4b). Only one was downregulated in serum and in eutopic endometrium during both the proliferative and secretory phases.

## 4. Discussion

Endometriosis is an enigmatic disease because of its unknown etiology. The need for laparoscopy surgery to diagnose endometriosis leads to a considerable delay before medical or surgical treatment can be undertaken, thus justifying the crucial need for diagnostic markers [17]. Moreover, endometrial biopsies are minimally invasive compared to laparoscopy surgery, although not particularly pleasant or comfortable for women [16,21]. In such a context, high-quality omics analyses appear highly relevant and important as a prelude to the in vivo testing of hypotheses based on the obtained data. As already mentioned, the most widely accepted theory concerning the origin of endometriosis is based on cell invasion following retrograde menstruation (i.e., Sampson’s theory [8,9]). Indeed, this suggests that the eutopic endometrium itself could play a role in the establishment of the disease. Thus, we conducted a large-scale differential proteomics analysis with no a priori hypothesis on eutopic endometrium from women with endometriosis vs. eutopic endometrium from women without. Our aim was to identify differentially expressed proteins that could be proposed as potential biological markers of the disease. 

Our control population constituted a highly homogeneous group, as shown by PCA (Figure 1b), underlining the importance of maintaining strict inclusion/exclusion criteria [37]. At the global proteome scale, we could clearly separate control from endometriosis samples (Figure 1b). The homogeneity might be due to a statistical error arising from the small number of patients, which is why this observation should be confirmed with larger sample cohorts. Indeed, the main limitation of the present study is the small size of the cohort involved (five controls and four endometriosis), although well-defined by specialist surgeons. In contrast to the control group, our patient population was heterogeneous, as also shown on PCA graphs (Figure 1b,c), highlighting the variability of the disease. It is important to stress that endometriosis samples were obtained from women with no hormonal treatment, which is rare and difficult to obtain as symptomatic endometriosis-suspected patients are immediately administered with oestro progestatives. That explains the small size of our cohort. 

Among the 5301 identified proteins, 543 were differentially expressed in the eutopic endometrium of women with endometriosis. KEGG interrogation allowed the identification of two enriched pathways: the PI3K/AKT signaling pathway and focal adhesion. The PI3K/AKT signaling pathway appears to be enriched in our upregulated proteins. This pathway regulates fundamental cellular functions, such as cell proliferation, cell growth, and apoptosis in response to many types of stimuli (inflammation, toxicants, etc.) [51]. In endometriosis, some studies, including our own, suggest upregulation of AKT1, which may cause induction of the AKT signaling pathway. It is possible that this is involved in the establishment of ectopic lesions through retrograde menstruation, as previously described [52,53]. Moreover, upregulation of the AKT pathway could affect the decidualization process and may thus be unfavorable for embryo implantation [54,55] and negatively affect fertility in women with endometriosis. Indeed, the endometrial biopsies used in our study were performed during the putative window of implantation.

Focal adhesion is a pathway, which plays essential roles in cell motility, cell proliferation, cell differentiation, etc. It is also known to allow communication between the extracellular matrix (ECM) and cytoskeleton through specific ligands of the ECM, such as laminins, and the integrin receptor [56]. Laminins are heterotrimeric proteins composed of alpha, beta, and gamma chains, each encoded by distinct genes. In our study, we found some members of the laminin family to be upregulated in eutopic endometrium, i.e., laminin subunit alpha-2 (LAMA2), laminin subunit alpha-5 (LAMA5), laminin subunit beta-1 (LAMB1), laminin subunit beta-2 (LAMB2), laminin subunit gamma-3 (LAMC3), and laminin subunit gamma-1 (LAMC1). (Supplemental Table S1). Some laminins have already been shown in the literature to be associated with endometriosis. For example, according to a case/control study involving 60 women with endometriosis and 20 controls, serum levels of laminin-1 had a sensitivity of 72% and a specificity of 68% for the diagnosis of stage II-III-IV endometriosis [57]. In a previous study, Laudanski et al. evaluated LAMA5 levels in eutopic endometrium during the proliferative phase and showed significantly higher levels of the protein in women with moderate/severe (III/IV) endometriosis [58]. More recently, another study concluded that there is an association of a LAMA5 (rs2427284) SNP with advanced stages of endometriosis (III/IV) [59]. Both studies made similar conclusions and suggested that laminins could be involved in the development of endometriosis, especially in adhesion, migration, and invasion. Although the dysregulation of several Integrin subunits has been observed in endometriosis [21], our results show, in a complementary way, that the integrin beta-4 (ITGB4) receptor for laminin and integrin alpha-IIb (ITGA2B) were upregulated in the eutopic endometrium of women with endometriosis. (Supplemental Table S1).

Focal adhesion kinase 1 (FAK1), another key actor of the focal adhesion pathway, was overexpressed in the endometriosis samples. FAK has already been reported to be upregulated in eutopic endometrium in the context of endometriosis [60], especially during the secretory phase [61], suggesting that the protein may be involved in the pathogenesis and development of the disease. Altogether, these results reinforce the idea that the eutopic endometrium may be involved in the apparition of peritoneal endometriotic lesions, corresponding to Sampson’s theory. 

Here, only 24 proteins were found to be downregulated. Given the small number, only gene ontology enrichment analysis could be performed, showing that these downregulated proteins are mainly involved in the immune response (Figure 2), especially neutrophil activation. Several members of the S100 protein family were under-expressed in the eutopic endometrium of women with endometriosis during the mid-secretory phase: S100A7, S100A8, S100A9, and S100A12. This protein family consists of 24 members with various functions and may be involved in cell proliferation, differentiation, inflammation, migration, etc. In recent years, the S100 protein family has attracted attention in the field of biomarker research, as members have been shown to be associated with inflammatory diseases and cancer [62]. Interestingly, a recent review proposed targeted mass spectrometry analysis as a viable approach to monitor and target these proteins [63].

S100A7 has been described in breast cancer [62] but the present study is the first to highlight a potential link between S100A7 and endometriosis. S100A8 and S100A9 can form homodimers or heterodimers (S100A8/A9) and both S100A8, S100A9, and S100A8/A9 stimulate neutrophils and are involved in neutrophil migration [64]. S100A8 has been shown to be significantly more abundant in the peritoneal fluid of women with deep endometriosis (stage III-IV of the rASRM classification) during the early stage of the disease (I-II) [65]. An increase in neutrophil granulocytes has also been observed in the peritoneal fluid of women with endometriosis, suggesting it may play an important role in the formation of early lesions in the peritoneal cavity [66].

Although not identified in our list of differentially expressed proteins, other members of the S100 protein family have been shown to play a crucial role in the receptivity of the mid-secretory endometrium, such as S100A10 [67]. The S100P protein displays high levels of expression in the eutopic endometrium during the window of implantation [68]. Additionally, decreased expression of S100A11 may affect endometrial receptivity and embryo implantation [69]. We identified S100A10, S100A11, and S100P but they did not show differential expression. Given that the women of the control population underwent laparoscopy for unexplained infertility, we were unable to draw any conclusions on the potential impact of endometriosis on endometrial receptivity and implantation failure. This needs to be further evaluated through additional targeted studies with samples from disease-free fertile women as the control population. 

In the second part of our study, we integrated our data with those from a previous study [35], as it had similar inclusion and exclusion criteria for patients with endometriosis and the control population, i.e., surgical proof of the presence or absence of endometriosis. The main difference between the two studies was the phase of the menstrual cycle; we focused on the secretory phase, whereas Manousopoulou et al. studied the proliferative phase. The authors used an LC-MS/MS and iTRAQ approach to find differentially expressed proteins in the eutopic endometrium (8 vs. 8) and serum (4 vs. 4) of women with or without endometriosis. This allowed the identification of 1,214 differentially expressed proteins in the eutopic endometrium and 404 in the serum. Although the sampling and the method can influence the result, it is primarily the false discovery rate of <5% used by the authors that leads to the higher number of proteins identified in the Manousopoulou study than in ours. For the mass-spectrometry analyses, we used more stringent criteria for protein identification with an FDR set to 1% at the protein level. 

Eleven proteins from our study and that of Manousopoulou et al. showed the same trend of dysregulation in the eutopic endometrium, regardless of the phase of the menstrual cycle and 16, including one laminin, were identified as being overexpressed in both the serum and eutopic endometrium of women with endometriosis (Table 2). However, there was no significant enrichment in functional annotations. Eight downregulated proteins are common to datasets from the two studies. Among them, the downregulated expression of lactotransferrin in the endometrium was confirmed for endometriosis, independently of the phase of the menstrual cycle. Expression of this protein was also downregulated in the serum. Lactotransferrin is used as a marker of neutrophil granulocyte activation and was shown to be present at lower concentrations in the peritoneal fluid of women with minimal endometriosis (stage I of rASRM classification) [70,71]. Another protein of interest is cathepsin G, of which the expression is downregulated not only in the endometrium in the secretory phase of women with endometriosis but also in their serum. These results are not in accordance with a previous study showing that the cathepsin G concentration was higher in the eutopic endometrium from women with endometriosis [72,73] but during the proliferative phase of the cycle. The different phases of the cycle studied may explain the differences observed between our study and that of Manousopoulou et al. and earlier studies. In total, six proteins were downregulated in the eutopic endometrium during both the proliferative and secretory phases: S100A8, S100A9, S100A12, platelet factor 4, platelet basic protein, and serum amyloid P-component. As already mentioned, the three S100 proteins are involved in neutrophil-mediated immunity and could therefore be considered for further in-depth analyses.

## 5. Conclusions

Our study corresponds to the preclinical discovery phase of endometriosis biomarkers [16] and underlines potential promising protein candidates for endometriosis screening. Here, we adopted a shotgun proteomics strategy and used a new generation of mass spectrometer (Tims TOF Pro; Bruker Daltonics) that offers unprecedented sensitivity and resolution for analyzing complex proteomes. 

This study also provides additional data to explain the etiology of peritoneal endometriosis, underlying the importance of cell adhesion and the immune response in eutopic endometrial cells and the apparition of the disease. Further research is also needed to confirm our results on a larger cohort of endometriosis samples. 

Overall, our results provide new insights into the mechanisms underlying endometriosis and its etiology and constitute a first step towards the development of non-invasive diagnostic biomarkers for this disease.

## Figures and Tables

**Table 1:** Inclusion and exclusion criterion of patients and controls.

	Inclusion Criterion	Exclusion Criterion
♀ ⟪endometriosis⟫ N = 4	Endometriosis (with symptoms) Histologic confirmation of endometriosis No hormonal treatment Laparoscopy between day 19 and 24 of menstrual cycle Presence of superficial and active lesions with angiogenesis around Presence of characteristic lesions in Pouch of Douglas	Adenomyosis No symptom Hormonal treatment Other day of menstrual cylce Hydrosalpinx
♀ ⟪control⟫ N = 5	Underwent surgery for infertility No pain symptom No hormonal treatment Laparoscopy between day 19 and 24 of menstrual cycle No visible lesion with ultrasound No cyst	Hydrosalpinx Endometriosis or other disease with effect on endometrium Hormonal treatment Other day of menstrual cycle

**Table 2:** Proteins found to be significantly up- or down-regulated in eutopic endometrium from controls compared with that from endometriosis patients.

UniProt iD.	Protein Names	Gene Names	Méar et al. Endometrium (Secretory)	Manousopoulou et al. Endometrium (Proliferative)	Manousopoulou et al. Serum
Q9Y446	Plakophilin-3	PKP3	up	up	-
A0AV96	RNA-binding protein 47	RBM47	up	up	-
Q9P2M7	Cingulin	CGN	up	up	-
O95210	Starch-binding domain-containing protein 1	STBD1	up	up	-
Q9UIL1	Short coiled-coil protein	SCOC	up	up	-
O00292	Left-right determination factor 2	LEFTY2	up	up	-
P13688	Carcinoembryonic antigen-related cell adhesion molecule 1	CEACAM1	up	up	-
P22748	Carbonic anhydrase 4	CA4	up	up	-
Q06710	Paired box protein Pax-8	PAX8	up	up	-
Q12974	Protein tyrosine phosphatase type IVA 2	PTP4A2	up	up	-
Q76KP1	N-acetyl-beta-glucosaminyl-glycoprotein 4-beta-N-acetylgalactosaminyltransferase 1	B4GALNT4	up	up	-
P11047	Laminin subunit gamma-1	LAMC1	up	-	up
Q14203	Dynactin subunit 1	DCTN1	up	-	up
Q6IBS0	Twinfilin-2	TWF2	up	-	up
O00429	Dynamin-1-like protein	DNM1L	up	-	up
Q15942	Zyxin	ZYX	up	-	up
Q14247	Src substrate cortactin	CTTN	up	-	up
P12081	Histidine—tRNA ligase, cytoplasmic	HARS	up	-	up
O14974	Protein phosphatase 1 regulatory subunit 12A	PPP1R12A	up	-	up
Q27J81	Inverted formin-2	INF2	up	-	up
P14317	Hematopoietic lineage cell-specific protein	HCLS1	up	-	up
O43294	Transforming growth factor beta-1-induced transcript 1 protein	TGFB1I1	up	-	up
Q99426	Tubulin-folding cofactor B	TBCB	up	-	up
O95747	Serine/threonine-protein kinase OSR1	OXSR1	up	-	up
Q05209	Tyrosine-protein phosphatase non-receptor type 12	PTPN12	up	-	up
Q8N392	Rho GTPase-activating protein 18	ARHGAP18	up	-	up
P98196	Probable phospholipid-transporting ATPase IH	ATP11A	up	-	up
P08311	Cathepsin G	CTSG	down	-	down
P06702	Protein S100-A9	S100A9	down	down	-
P05109	Protein S100-A8	S100A8	down	down	-
P02775	Platelet basic protein	PPBP	down	down	-
P80511	Protein S100-A12	S100A12	down	down	-
P02743	Serum amyloid P-component	APCS	down	down	-
P02776	Platelet factor 4	PF4	down	down	-
P02788	Lactotransferrin	LTF	down	down	down

## Data Availability

The mass spectrometry proteomics data have been deposited at the ProteomeXchange Consortium via the PRIDE (Perez-Riverol et al. 2019) partner repository with the dataset identifier PXD012981 and 10.6019/PXD012981.

## Supplementary Materials

The following supporting information can be downloaded at: www.mdpi.com/article/10.3390/diagnostics12020419/s1, Supplemental Table S1. List of differentially expressed proteins in control versus endometriosis at the tissue level (.xlsx). Mass Spectrometry proteomics data. PXD012981 (ProteomeXchange Consortium).

## References

[1] Giudice L.C., Kao L.C. (2004). Endometriosis. Lancet.

[2] Zondervan K.T., Becker C.M., Koga K., Missmer S.A., Taylor R.N., Viganò P. (2018). Endometriosis. Nat. Rev. Dis. Primers.

[3] Nisolle M., Donnez J. (1997). Peritoneal Endometriosis, Ovarian Endometriosis, and Adenomyotic Nodules of the Rectovaginal Septum Are Three Different Entities. Fertil. Steril..

[4] Nezhat C., Nezhat F., Nezhat C. (2012). Endometriosis: Ancient Disease, Ancient Treatments. Fertil. Steril..

[5] Vinatier D., Orazi G., Cosson M., Dufour P. (2001). Theories of Endometriosis. Eur. J. Obs. Gynecol. Reprod. Biol..

[6] Vercellini P., Viganò P., Somigliana E., Fedele L. (2014). Endometriosis: Pathogenesis and Treatment. Nat. Rev. Endocrinol..

[7] Bulun S.E. (2009). Endometriosis. N. Engl. J. Med..

[8] Sampson J.A. (1922). Ovarian Hematomas of Endometrial Type (Perforating Hemorrhagic Cysts of the Ovary) and Implantation Adenomas of Endometrial Type. Boston Med. Surg. J..

[9] Sampson J.A. (1927). Metastatic or Embolic Endometriosis, Due to the Menstrual Dissemination of Endometrial Tissue into the Venous Circulation. Am. J. Pathol..

[10] D’Hooghe T.M., Debrock S. (2002). Endometriosis, Retrograde Menstruation and Peritoneal Inflammation in Women and in Baboons. Hum. Reprod. Update.

[11] Koninckx P.R., Ussia A., Adamyan L., Wattiez A., Gomel V., Martin D.C. (2019). Pathogenesis of Endometriosis: The Genetic/Epigenetic Theory. Fertil. Steril..

[12] Ramin-Wright A., Schwartz A.S.K., Geraedts K., Rauchfuss M., Wölfler M.M., Haeberlin F., von Orelli S., Eberhard M., Imthurn B., Imesch P. (2018). Fatigue—A Symptom in Endometriosis. Hum. Reprod..

[13] Ballard K., Lowton K., Wright J. (2006). What’s the Delay? A Qualitative Study of Women’s Experiences of Reaching a Diagnosis of Endometriosis. Fertil. Steril..

[14] Hudelist G., Fritzer N., Thomas A., Niehues C., Oppelt P., Haas D., Tammaa A., Salzer H. (2012). Diagnostic Delay for Endometriosis in Austria and Germany: Causes and Possible Consequences. Hum. Reprod..

[15] Staal A.H.J., van der Zanden M., Nap A.W. (2016). Diagnostic Delay of Endometriosis in the Netherlands. Gynecol. Obs. Investig..

[16] Fassbender A., Burney R.O., O D.F., D’Hooghe T., Giudice L. (2015). Update on Biomarkers for the Detection of Endometriosis. Biomed. Res. Int..

[17] Rogers P.A.W., Adamson G.D., Al-Jefout M., Becker C.M., D’Hooghe T.M., Dunselman G.A.J., Fazleabas A., Giudice L.C., Horne A.W., Hull M.L. (2017). Research Priorities for Endometriosis. Reprod. Sci..

[18] Bergman-Larsson J., Gustafsson S., Méar L., Huvila J., Tolf A., Olovsson M., Pontén F., Edqvist P.-H.D. (2022). Combined Expression of HOXA11 and CD10 Identifies Endometriosis versus Normal Tissue and Tumors. Ann. Diagn. Pathol..

[19] Bedaiwy M.A., Falcone T. (2004). Laboratory Testing for Endometriosis. Clin. Chim. Acta.

[20] May K.E., Conduit-Hulbert S.A., Villar J., Kirtley S., Kennedy S.H., Becker C.M. (2010). Peripheral Biomarkers of Endometriosis: A Systematic Review. Hum. Reprod. Update.

[21] May K.E., Villar J., Kirtley S., Kennedy S.H., Becker C.M. (2011). Endometrial Alterations in Endometriosis: A Systematic Review of Putative Biomarkers. Hum. Reprod. Update.

[22] Acién P., Velasco I. (2013). Endometriosis: A Disease That Remains Enigmatic. ISRN Obs. Gynecol..

[23] Gupta D., Hull M.L., Fraser I., Miller L., Bossuyt P.M.M., Johnson N., Nisenblat V. (2016). Endometrial Biomarkers for the Non-Invasive Diagnosis of Endometriosis. Cochrane Database Syst. Rev..

[24] Nisenblat V., Bossuyt P.M.M., Shaikh R., Farquhar C., Jordan V., Scheffers C.S., Mol B.W.J., Johnson N., Hull M.L. (2016). Blood Biomarkers for the Non-Invasive Diagnosis of Endometriosis. Cochrane Database Syst. Rev..

[25] Ferrero S., Gillott D.J., Remorgida V., Ragni N., Venturini P.L., Grudzinskas J.G. (2008). Proteomics Technologies in Endometriosis. Expert. Rev. Proteom..

[26] Siva A.B., Srivastava P., Shivaji S. (2014). Understanding the Pathogenesis of Endometriosis through Proteomics: Recent Advances and Future Prospects. Proteom. Clin. Appl..

[27] Ferrero S. (2019). Proteomics in the Diagnosis of Endometriosis: Opportunities and Challenges. Proteom. Clin. Appl..

[28] Ferrero S., Gillott D.J., Remorgida V., Anserini P., Leung K.-Y., Ragni N., Grudzinskas J.G. (2007). Proteomic Analysis of Peritoneal Fluid in Women with Endometriosis. J. Proteome Res..

[29] Ten Have S., Fraser I., Markham R., Lam A., Matsumoto I. (2007). Proteomic Analysis of Protein Expression in the Eutopic Endometrium of Women with Endometriosis. Proteom. Clin. Appl..

[30] El-Kasti M.M., Wright C., Fye H.K.S., Roseman F., Kessler B.M., Becker C.M. (2011). Urinary Peptide Profiling Identifies a Panel of Putative Biomarkers for Diagnosing and Staging Endometriosis. Fertil. Steril..

[31] Kyama C.M., Mihalyi A., Gevaert O., Waelkens E., Simsa P., Van de Plas R., Meuleman C., De Moor B., D’Hooghe T.M. (2011). Evaluation of Endometrial Biomarkers for Semi-Invasive Diagnosis of Endometriosis. Fertil. Steril..

[32] Fassbender A., Verbeeck N., Börnigen D., Kyama C.M., Bokor A., Vodolazkaia A., Peeraer K., Tomassetti C., Meuleman C., Gevaert O. (2012). Combined MRNA Microarray and Proteomic Analysis of Eutopic Endometrium of Women with and without Endometriosis. Hum. Reprod..

[33] Williams K.E., Miroshnychenko O., Johansen E.B., Niles R.K., Sundaram R., Kannan K., Albertolle M., Zhou Y., Prasad N., Drake P.M. (2015). Urine, Peritoneal Fluid and Omental Fat Proteomes of Reproductive Age Women: Endometriosis-Related Changes and Associations with Endocrine Disrupting Chemicals. J. Proteom..

[34] Grande G., Vincenzoni F., Milardi D., Pompa G., Ricciardi D., Fruscella E., Mancini F., Pontecorvi A., Castagnola M., Marana R. (2017). Cervical Mucus Proteome in Endometriosis. Clin. Proteom..

[35] Manousopoulou A., Hamdan M., Fotopoulos M., Garay-Baquero D.J., Teng J., Garbis S.D., Cheong Y. (2019). Integrated Eutopic Endometrium and Non-Depleted Serum Quantitative Proteomic Analysis Identifies Candidate Serological Markers of Endometriosis. Proteom. Clin. Appl..

[36] Fowler P.A., Tattum J., Bhattacharya S., Klonisch T., Hombach-Klonisch S., Gazvani R., Lea R.G., Miller I., Simpson W.G., Cash P. (2007). An Investigation of the Effects of Endometriosis on the Proteome of Human Eutopic Endometrium: A Heterogeneous Tissue with a Complex Disease. Proteomics.

[37] Greaves E., Critchley H.O.D., Horne A.W., Saunders P.T.K. (2017). Relevant Human Tissue Resources and Laboratory Models for Use in Endometriosis Research. Acta Obs. Gynecol. Scand..

[38] Becker C.M., Laufer M.R., Stratton P., Hummelshoj L., Missmer S.A., Zondervan K.T., Adamson G.D. (2014). WERF EPHect Working Group World Endometriosis Research Foundation Endometriosis Phenome and Biobanking Harmonisation Project: I. Surgical Phenotype Data Collection in Endometriosis Research. Fertil. Steril..

[39] Fassbender A., Rahmioglu N., Vitonis A.F., Viganò P., Giudice L.C., D’Hooghe T.M., Hummelshoj L., Adamson G.D., Becker C.M., Missmer S.A. (2014). World Endometriosis Research Foundation Endometriosis Phenome and Biobanking Harmonisation Project: IV. Tissue Collection, Processing, and Storage in Endometriosis Research. Fertil. Steril..

[40] Rahmioglu N., Fassbender A., Vitonis A.F., Tworoger S.S., Hummelshoj L., D’Hooghe T.M., Adamson G.D., Giudice L.C., Becker C.M., Zondervan K.T. (2014). World Endometriosis Research Foundation Endometriosis Phenome and Biobanking Harmonization Project: III. Fluid Biospecimen Collection, Processing, and Storage in Endometriosis Research. Fertil. Steril..

[41] Vitonis A.F., Vincent K., Rahmioglu N., Fassbender A., Buck Louis G.M., Hummelshoj L., Giudice L.C., Stratton P., Adamson G.D., Becker C.M. (2014). World Endometriosis Research Foundation Endometriosis Phenome and Biobanking Harmonization Project: II. Clinical and Covariate Phenotype Data Collection in Endometriosis Research. Fertil. Steril..

[42] Liu E., Nisenblat V., Farquhar C., Fraser I., Bossuyt P.M.M., Johnson N., Hull M.L. (2015). Urinary Biomarkers for the Non-Invasive Diagnosis of Endometriosis. Cochrane Database Syst. Rev..

[43] Melaine N., Com E., Bellaud P., Guillot L., Lagarrigue M., Morrice N.A., Guével B., Lavigne R., Velez de la Calle J.-F., Dojahn J. (2018). Deciphering the Dark Proteome: Use of the Testis and Characterization of Two Dark Proteins. J. Proteome Res..

[44] Jumeau F., Com E., Lane L., Duek P., Lagarrigue M., Lavigne R., Guillot L., Rondel K., Gateau A., Melaine N. (2015). Human Spermatozoa as a Model for Detecting Missing Proteins in the Context of the Chromosome-Centric Human Proteome Project. J. Proteome Res..

[45] Colinet H., Pineau C., Com E. (2017). Large Scale Phosphoprotein Profiling to Explore Drosophila Cold Acclimation Regulatory Mechanisms. Sci. Rep..

[46] Fermin D., Basrur V., Yocum A.K., Nesvizhskii A.I. (2011). Abacus: A Computational Tool for Extracting and Pre-Processing Spectral Count Data for Label-Free Quantitative Proteomic Analysis. Proteomics.

[47] R Core Team R: A Language and Environment for Statistical Computing. http://r.meteo.uni.wroc.pl/web/packages/dplR/vignettes/intro-dplR.pdf.

[48] Lê S., Josse J., Husson F. (2008). FactoMineR: An R Package for Multivariate Analysis. J. Stat. Softw..

[49] Combes F., Loux V., Vandenbrouck Y. (2021). GO Enrichment Analysis for Differential Proteomics Using ProteoRE. Methods Mol. Biol..

[50] Bardou P., Mariette J., Escudié F., Djemiel C., Klopp C. (2014). Jvenn: An Interactive Venn Diagram Viewer. BMC Bioinform..

[51] Kim J.J., Yin X. (2011). Signaling Pathways in Endometriosis (Eutopic/Ectopic). Endometriosis.

[52] Laudanski P., Szamatowicz J., Kowalczuk O., Kuźmicki M., Grabowicz M., Chyczewski L. (2009). Expression of Selected Tumor Suppressor and Oncogenes in Endometrium of Women with Endometriosis. Hum. Reprod..

[53] Kim T.H., Yu Y., Luo L., Lydon J.P., Jeong J.-W., Kim J.J. (2014). Activated AKT Pathway Promotes Establishment of Endometriosis. Endocrinology.

[54] Yin X., Pavone M.E., Lu Z., Wei J., Kim J.J. (2012). Increased Activation of the PI3K/AKT Pathway Compromises Decidualization of Stromal Cells from Endometriosis. J. Clin. Endocrinol. Metab..

[55] Lessey B.A., Kim J.J. (2017). Endometrial Receptivity in the Eutopic Endometrium of Women with Endometriosis: It Is Affected, and Let Me Show You Why. Fertil. Steril..

[56] Romer L.H., Birukov K.G., Garcia J.G.N. (2006). Focal Adhesions: Paradigm for a Signaling Nexus. Circ. Res..

[57] Ozhan E., Kokcu A., Yanik K., Gunaydin M. (2014). Investigation of Diagnostic Potentials of Nine Different Biomarkers in Endometriosis. Eur. J. Obs. Gynecol. Reprod. Biol..

[58] Laudanski P., Charkiewicz R., Kuzmicki M., Szamatowicz J., Świątecka J., Mroczko B., Niklinski J. (2014). Profiling of Selected Angiogenesis-Related Genes in Proliferative Eutopic Endometrium of Women with Endometriosis. Eur. J. Obs. Gynecol. Reprod. Biol..

[59] Christofolini D.M., Mafra F.A., Catto M.C., Bianco B., Barbosa C.P. (2019). New Candidate Genes Associated to Endometriosis. Gynecol. Endocrinol..

[60] Mu L., Ma Y.-Y. (2015). Expression of Focal Adhesion Kinase in Endometrial Stromal Cells of Women with Endometriosis Was Adjusted by Ovarian Steroid Hormones. Int. J. Clin. Exp. Pathol..

[61] Mu L., Zheng W., Wang L., Chen X.-J., Zhang X., Yang J.-H. (2008). Alteration of Focal Adhesion Kinase Expression in Eutopic Endometrium of Women with Endometriosis. Fertil. Steril..

[62] Donato R., Cannon B.R., Sorci G., Riuzzi F., Hsu K., Weber D.J., Geczy C.L. (2013). Functions of S100 Proteins. Curr. Mol. Med..

[63] Martínez-Aguilar J., Molloy M.P. (2019). Targeted Mass Spectrometry of S100 Proteins. Methods Mol. Biol..

[64] Ryckman C., Vandal K., Rouleau P., Talbot M., Tessier P.A. (2003). Proinflammatory Activities of S100: Proteins S100A8, S100A9, and S100A8/A9 Induce Neutrophil Chemotaxis and Adhesion. J. Immunol..

[65] Ferrero S., Gillott D.J., Remorgida V., Anserini P., Ragni N., Grudzinskas J.G. (2008). Peritoneal Fluid Proteome in Women with Different ASRM Stages of Endometriosis. Gynecol. Endocrinol..

[66] Symons L.K., Miller J.E., Kay V.R., Marks R.M., Liblik K., Koti M., Tayade C. (2018). The Immunopathophysiology of Endometriosis. Trends Mol. Med..

[67] Bissonnette L., Drissennek L., Antoine Y., Tiers L., Hirtz C., Lehmann S., Perrochia H., Bissonnette F., Kadoch I.-J., Haouzi D. (2016). Human S100A10 Plays a Crucial Role in the Acquisition of the Endometrial Receptivity Phenotype. Cell Adh. Migr..

[68] Tong X.-M., Lin X.-N., Song T., Liu L., Zhang S.-Y. (2010). Calcium-Binding Protein S100P Is Highly Expressed during the Implantation Window in Human Endometrium. Fertil. Steril..

[69] Liu X.-M., Ding G.-L., Jiang Y., Pan H.-J., Zhang D., Wang T.-T., Zhang R.-J., Shu J., Sheng J.-Z., Huang H.-F. (2012). Down-Regulation of S100A11, a Calcium-Binding Protein, in Human Endometrium May Cause Reproductive Failure. J. Clin. Endocrinol. Metab..

[70] Polak G., Wertel I., Tarkowski R., Morawska D., Kotarski J. (2007). Decreased Lactoferrin Levels in Peritoneal Fluid of Women with Minimal Endometriosis. Eur. J. Obs. Gynecol. Reprod. Biol..

[71] Riley C.F., Moen M.H., Videm V. (2007). Inflammatory Markers in Endometriosis: Reduced Peritoneal Neutrophil Response in Minimal Endometriosis. Acta Obs. Gynecol. Scand..

[72] Laudanski P., Gorodkiewicz E., Ramotowska B., Charkiewicz R., Kuzmicki M., Szamatowicz J. (2013). Determination of Cathepsins B, D and G Concentration in Eutopic Proliferative Endometrium of Women with Endometriosis by the Surface Plasmon Resonance Imaging (SPRI) Technique. Eur. J. Obs. Gynecol. Reprod. Biol..

[73] Grzywa R., Gorodkiewicz E., Burchacka E., Lesner A., Laudański P., Łukaszewski Z., Sieńczyk M. (2014). Determination of Cathepsin G in Endometrial Tissue Using a Surface Plasmon Resonance Imaging Biosensor with Tailored Phosphonic Inhibitor. Eur. J. Obs. Gynecol. Reprod. Biol..

